# RIGHT-SIDED BOCHDALEK HERNIA IN ADULT ASSOCIATED WITH CHOLESTATIC SYNDROME: CASE REPORT

**DOI:** 10.1590/S0102-6720201500030023

**Published:** 2015

**Authors:** José Moreira dos SANTOS-NETTO, Cássio Virgílio Cavalcante OLIVEIRA, Marcelo Gonçalves SOUSA

**Affiliations:** Department of Surgery, Lauro Wanderley University Hospital, João Pessoa, PB, Brazil.

## INTRODUCTION

The congenital diaphragmatic hernia (CDH) is defined as an anatomical defect on diaphragm, which permits the herniation of abdominal viscera into the thorax^4^. The hernia occurs due to an incomplete occlusion of the pleuroperitoneal channel during the embrionary period. The main cause of the incomplete closure can be a genetic mutation, a teratogen or both.

In terms of anatomic location, the CDH can be classified as Bochdalek type when an incomplete pleuroperitoneal channel occlusion is found posterolaterally; as Morganni type, while the defect is seen retrosternally; and yet as a congenital transhiatal esophagic type hernia. Among them, the Bochdalek type is the most common, found in 78-90% of patients; the Morganni type, in 1,5-6% of cases; and transhiatal, 14-24%^11^.

In most cases, the clinical impact occurs in the neonatal period, since only 10% of hernias are diagnosed after this period^7^. In neonates, the clinical presentation is acute, providing a higher morbidity and mortality. In adulthood, symptoms, if any, are more insidious, vague and intermittent, affecting not only the pulmonary dynamics, but also the gastrointestinal function^5^.

Chest X-ray and CT scan may be used^2^^,^ 8 ^,^ 12.Nevertheless, CDH findings are incidental when performing radiological examinations for other reasons, with the right-sided Bochdalek hernia accounting for 68% of cases.^9^^,^ 13

In elective situations, the minimally invasive surgery, either via laparoscopic or thoracoscopic can be used, but with limited application in cases of right-sided hernia^3^^,^ 8. Minor defects, technically easier to fix, can be sutured normally; in the case of larger apertures, or even hemidiaphragmatic agenesis, the use of nonabsorbable polypropylene mesh is the only solution.

## CASE REPORT

A forty-five-year-old woman, caucasian, married, admitted into our service, complains of insidious jaundice in the previous five months, associated with itching, choluria and abdominal distension. Eight months before, the patient had a spontaneous abortion during the fourth month of pregnancy. Reported history of cranial malformation at birth, duly corrected surgically.

The patient was underwent to serological laboratory tests for viral hepatitis, resulting all negative. The serum total bilirubin was 4.3 mg/dl due to direct fraction, and creatinine was 1.7 mg/dl. During ultrasound exam, it was shown dilatation of intrahepatic bile ducts and liver enlargement, mainly of the right hepatic lobe. Computed tomography with contrast revealed the partial absence of diaphragmatic dome in the right posterolateral portion, showing an herniation of the right hepatic lobe, the right kidney and right adrenal gland, associated with marked atrophy of the left hepatic lobe and right pulmonary hypoplasia.

**FIGURE 1:**
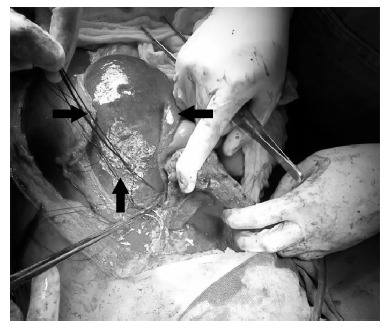
- Intraoperatory registry of the diaphragmatic opening (among black arrows)

The patient was initially submitted to laparotomy which showed massive hepatomegaly due to the right lobe, whose edge was at the level of the umbilicus scar. Due to the great technical difficulties, thoracophrenolaparotomy was conducted, which showed failure at right diaphragm dome of approximately 10 cm (Figure 1), herniation of the hepatic lobe, kidney, right adrenal, colon, associated to a partial twist of the common bile duct and dilatation of the biliary tract upstream and excessive lateral traction of first and second portions of duodenum, and of the head of the pancreas.

Posteriorly, the reduction of the hernia contents back into the abdominal cavity was done, with subsequent apposition of polypropylene mesh over the hernia defect and drainage of the chest. Intraoperative cholangiography was performed demonstrating recanalization of the bile duct and satisfying contrast escape into the duodenum.

The postoperative period went on with normalization of bilirubin and renal function. The patient died weeks later due to nosocomial pneumonia and sepsis during hospitalization in intensive care unit.

## DISCUSSION

The CDH incidence in general population varies between 2.5-3.8 cases per 10,000 births. There is some difficulty in establishing the prevalence of herniated Bochdalek in adults. A retrospective study of more than 13,000 CT scans of the abdomen showed a prevalence of about 0.17%. Other studies, however, agree with a greater prevalence, at about 6-12%, when computed tomography multislice is used^9^^,^ 13.

Studies have found that there is a lower risk of afrodescedent population being CDH carrier, compared to non-hispanic caucasians. There is a relative risk of 50% higher in children of mothers aged over 35, compared to maternal age between 20-24 years^4^. In this case, the patient is a caucasian descendent; however, maternal age at birth was 23 years.

Retrospective study of 116 cases between 1991 and 2002 in Australia, found a prevalence of 46.6% of clinically significant abnormalities, and 38.8% minimum clinically significant abnormalities, being the most frequent neurological, musculoskeletal, dysmorphic, genitourinary and gastrointestinal^1^. In this report above, it was mentioned history of cranial malformation, unspecified by the patient.

The left-sided Bochdalek hernia is more prevalent than the right one, because the right-sided dome develops earlier and the liver avoids abdominal viscera protrusion^2^^,^ 11.However, hernias during the adulthood through the right dome are more frequent, appearing incidentally in 68% of diagnoses and affects mostly the females^13^.

The herniary defect varies from 1 cm of diameter until the complete absence of hemidiaphragm^8^. It was shown during the surgical procedure a diaphragmatic defect of approximately 10 cm in its largest diameter in the right posterolateral portion. In 20% of cases there is an hernial sac, in contrast to the majority of cases where there is a direct communication between the thoracic and abdominal cavities^8^. In 73% of cases, diaphragmatic hernia contains only visceral fat or omentum^7^. In the discussed case, it was not found an intraoperatively evidence of hernial sac.

Most CDH are diagnosed during the neonatal period, with only 10% of them discovered after this period^7^. The symptoms in adults are usually insidious and undefined. They can be not only gastrointestinal symptons, such as nausea, postprandial vomiting, abdominal pain, back pain, post-prandial bloating; but also respiratory complaints, such as dyspnea, chest pain, shoulder referred pain^5^^,^ 8. On physical examination, auscultation of typical bowel sounds of peristalsis is a specific signal for diaphragmatic hernia^2^.Specifically in this related case, the patient denied any symptoms throughout the life, looking for medical care because of a recent jaundice.

In distinction to the reality presented in this case, the most common acute complication, and the most feared, is the hernial incarceration and/or strangulation. The risk of strangulation in the right side is smaller, since the hernial orifice is generally larger than the contralateral side^5^. Some factors that increase intra-abdominal pressure, such as pregnancy, labor, coughing, sneezing and trauma may increase the risk of hernial content strangulation^5^^,^ 13. During the anamnesis of this patient, there are reports of pregnancy, with subsequent abortion three months before the beginning of cholestatic syndrome, a process that may have influenced the increase of the intra-abdominal pressure, twisting of the bile duct and appearance of jaundice.

Different modalities of diagnostic imaging can be used, among which chest x-ray, ultrasound, computadorized tomography, magnetic resonance. The sensitivity of chest x-ray is 70% and is not specific enough to exclude the diagnosis of Bochdalek hernia in case of negative result^2^^,^ 8. The gold standard for diagnosing is the double contrast tomography^2^. During the investigation of jaundice of the case in discussion, it was decided to request ultrasound and abdominal CT with contrast; after diagnosis, there was a complementation with chest tomography.

In urgent cases, the recommended treatment is open surgery with initial abdominal approach, applying for the thoracic via in cases of technical difficulty^5^^,^ 8. In elective situations, minimally invasive surgery, either via laparoscopic and/or videothoracoscopic can be used, but with limited application in cases of right-sided hernia^3^^,^ 8.

Minor defects, technically easier to fix, can be normally sutured; in situations of larger apertures, or even hemidiaphragmatic agenesis, the use of nonabsorbable mesh is the only way^2^. If large hernia has been reduced, the intra-abdominal pressure should be intensively monitored postoperatively in order to prevent the appearing of abdominal compartment syndrome^8^. The postoperative recurrence rate is considered rare^4^.
